# Thank You to Our Peer Reviewers in 2024

**DOI:** 10.1111/ene.70046

**Published:** 2025-01-21

**Didier Leys** (*Editor‐in‐Chief*), **Claudia Sommer** (*Deputy Editor*), **Barbara Borroni**, **Andrew Chan, Mark Edwards, Alessandro Tessitore, and Kristl Vonck** (*Associate Editors*), **Paulo Caramelli** and **Isabel Martins** (*Guest Editor*s)

Thank all the reviewers from around the world who have provided expert advice to ensure the publication of the best quality articles in the *European Journal of Neurology* and the journal of the *European Academy of Neurology*. Their feedback is crucial to improve the quality of manuscripts and of neurological care provided to patients. The names of those who reviewed manuscripts for the *European Journal of Neurology* in 2024 are listed below. Our warmest gratitude goes to all of them.

*indicates those who reviewed four manuscripts in 2024, **indicates those who reviewed five to eight, and ***indicates those who reviewed nine or more.

Aamodt, Anne Hege

Abbadessa, Gianmarco

Abdelhalk, Ahmed*

Abu‐Rumeileh, Samir

Accorroni, A.

Adhikary, Asit Baran

Afsin, Emine

Agarwal, Smriti

Aguglia, Umberto

Aguiar De Sousa, Diana

Aguirre‐Vidal, Yoshajandith

Ahmadizar, Fariba

Aktas, O.

Alberici, Antonella

Albo, Zimbul

Alboini, Paolo

Algoet, Chloé

Alhusaini, Saud

Allen, Jeffrey A.*

Almqvist, Tove

Alonso, Angelika

Alpkvist, Peter

Altomare, Daniele

Alves, Pedro

Al‐Zuhairy, Ali

Amaro, Sergio

Anagnostakou, Vania

Anagnostou, Evangelos

Andersen, Henning*

Anderson, Craig

Andrew, Melissa K.

Androdias, Géraldine

Angelini, Corrado

Appeltshauser, Luise

Araújo, Rui

Arba, Francesco

Arboix, Adria

Arsava, Ethem

Arshad, Qadeer

Arsovska, Anita

Ascherio, Alberto

Asseyer, Susanna

Aster, Hans‐Christoph

Attarian, Shahram**

Audebert, Heinrich

Audoin, Bertrand

Bain, Peter

Baizabal‐Carvallo, José

Baka, Panoraia

Balabanski, Anna

Balayssac, David

Baldelli, Luca

Ballester, Ly

Ballve, Alejandro

Banerjee, Soma

Bankstahl, Jens P.

Barber, Pa.

Barbera, Mariagnese

Barloese, Mads

Barnett, Carolina

Baron, Jean‐Claude

Batla, Amit

Baum, Petra

Bax, Francesco

Baykan‐Baykal, Betul

Beare, Richard

Beatriz Gago‐Veiga, Ana

Bede, Peter

Beier, Christoph

Beier, Dagmar

Béjot, Yannick

Ben Assayag, Einor

Benakis, Corinne

Benbadis, Selim

Ben‐Shabat, Ilan

Benussi, Alberto

Berciano, José

Bereczki, Dániel

Bergquist, Filip

Bernasconi, Neda

Berzero, Giulia

Bessi, Valentina

Beudel, Martijn

Bhaskar, Sonu

Bianchi, Alessia

Binarelli, Giulia

Biscetti, Leonardo

Bisdorff, Alexander

Bisecco, Alvino

Bittner, Stefan

Björkman‐Burtscher, Isabella

Blauenfeldt, Rolf

Blitshteyn, Svletana

Block, Valeria

Blystad, Ida

Bocci, Tommaso

Bodilsen, Jacob

Bohnen, Nicolaas

Bologna, Matteo

Boltze, Johannes

Bonanni, Paolo

Borger, Valeri

Bösel, Julia

Bouzigues, Arabella

Bove, R.

Bower, James

Braga‐Neto, Pedro

Brainin, Michael

Brandler, Ethan S. S.

Brandt, Roemer

Braschinsky, Mark

Brenner, David

Briani, Chiara

Brochet, Bruno

Brooks, David

Browland, Benjamin

Bruffaerts, Rose

Bsteh, Gabriel

Bu, Bitao

Budhram, Adrian

Buijink, Arthur W. G.

Bukhbinder, Avram

Burgunder, Jean‐Marc

Burt, R. K.

Byun, Eeeseung

Cagol, Alessandro

Caligiuri, Maria Eugenia

Calviere, Lionel

Calvo, Andrea

Camargos, Einstein

Campos‐Fernandez, Daniel

Cantero‐Fortiz, Yahveth

Canu, Elisa

Carcel, Cheryl

Carnes, Anna

Carotenuto, Antonio

Carrette, Sofie

Carson, Alan

Carvalho, Vanessa

Caso, Valeria

Castro, José

Cavaco, Sara

Cebulla, Nadine

Cecil, Kim

Cervenka, Mackenzie

Chabriat, Hugues

Chakraborty, Baidarbhi

Chanks, Hayley

Charidimou, Andreas

Cheng, Fubo

Chiang, Chia‐Chun

Chico‐García, Juan Luis

Chiò, Adriano

Chollet, François

Christensen, Hanne

Christoph, Stretz

Chu, Lily

Chung, Ki Wha

Chung, Seok Jong

Cimflova, Petra

Claeys, Kristl

Clottes, Paul

Cocito, Dario

Comi, Cristoforo

Comi, Giacomo

Consonni, M.

Conte, Antonella

Coon, Elizabeth

Cooper, Hannah

Coratti, Giorgia

Corboy, John R.

Corcia, Philippe

Costa, Rui Miguel

Coutinho, Jonathan

Cripe, Linda

Cruz‐Flores, Salvador

Cuadrado, Elisa

Cudkowicz, Merit

Czlonkowska, Anna

Dalli, Lachlan

Dalmau, Josep

Damato, Valentina

D'ambrosio, Alessandro

Dan, Gheorghe Andrei

Danhofer, Pavlina

Daniele, Antonio

Daumer, Martin

Davalos, Long

Davies, Benjamin

Davion, Jean‐Baptiste

Daying, Dai

De Boer, Sterre

De Boysson, Hubert

De Carvalho, Mamede**

De Lago, E.

De Los Reyes, Emily

De Mendonça, Alexandre

De Visser, Marianne***

De Waele, Liesbeth

Debnath, Monojit

Debs, Rabab

Defebvre, Luc

Degeneffe, Aurelie

Degirmenci, Yildiz

Deisenhammer, Florian

Del Brutto, Oscar

Della Pietra, Adriana*

Delmont, Emilien

Demeestere, Jelle

Deplanque, Dominique

Derex, Laurent

Dersch, Rick

Devalckeneer, Antoine

Devigili, Grazia

Di Lazzaro, Giulia

Di Lorenzo, Francesco

Di Tullio, Marco

Dichgans, Martin

Dickerson, Bradford C.

Ding, Y.H.**

Diomedi, Marina

Do, Thien

Doerrfuss, Jakob

Doneddu, Pietro

Donzuso, Giulia

Doppler, Kathrin**

Douros, Dr. Antonios

Drulovic, Jelena

Duarte, Christina

Dubbioso, Raffaele

Ducruet, Andrew F.

Durães, João

Dutra, L. A.

Edan, Gilles

Edlow, J. A.

Endo, Hironobu

Eriksson, Marie

Erken, Neziha

Esposito, Fabrizio

Ewers, M.

Fabbri, Margherita

Falcou, Anne

Fanciulli, Alessandra*

Fandler‐Höfler, Simon*

Fang, Fang

Farah, Jibril

Farah, Rita***

Fasano, Alfonso

Faught, E.

Fayaz, Alan

Fehlings, Michael

Ferini Strambi, Luigi

Fernandes, Carlos

Fernandez‐Ruiz, Javier

Ferrante, Enrico

Ferrari, Camilla

Ferrari‐Marinho, Taissa

Ferraro, Diana

Ferretti, Maria Teresa

Ferri, Raffaele

Ferro, José**

Festari, Cristina

Filho, Lúcio

Fingerle, Volker

Finitsis, Stephanos

Finsterer, Josef

Fionda, Laura

Fitzgerald, Julia

Fladt, Joachim

Flanagan, E.P.**

Flaus, Anthime

Fonseca, Ana Catarina

Fonseca, Ângelo

Fortanier, Etienne

Fox, Md

Foxe, David

Fracica, Elizabeth

Fragata, Isabel

Franca Jr., Marcondes

Freedman, Mark

Friedman, Daniel

Frol, Senta

Frosolini, Andrea

Fuentes, Blanca

Galgani, Alessandro

Galindo‐Ferreiro, Alicia

Gall, Seana

Gantenbein, Andreas R.

García Azorín, David

Garcia Pastor, Andres

Garcia‐Moreno, Hector

Garcin, Beatrice

Garibotto, Valentina

Gärtner, Jutta

Gaspard, N.

Ge, Yulin

Geerling, Joel

Geha, Paul

Geppetti, P.

Gewandter, Jennifer S.

Ghaderi, Sadegh

Ghelichi, Leila

Giedraitis, Vilmantas

Gilhus, Ne

Gilligan, Michael

Gilmour, Gabriela

Giorelli, Maurizio

Giovannoni, Gavin

Gissen, Paul

Giussani, Giorgia

Glass, Jonathan D.

Goadsby, Peter J.

Godasi, Raja

Goedee, H. Stephan

Golan, Daniel

Gonçalves, Natalia Gomes

Gonorazky, H.

Gouider, Riadh

Grach, Stephanie

Graus, Francesc

Graves, J. S.

Grigoriadis, Nikolaos

Grimm, Alexander

Grinberg, Lea

Grisold, Wolfgang

Groothuis, Jan

Grosu, Oxana

Grothe, Matthias

Guedj, Eric*

Guglielmetti, Monica

Guntinas‐Lichius, Orlando

Ha, Woo‐Seok

Habek, Mario

Hagemann, Anne

Hagenacker, Tim

Hahn, Katrin

Hale, David

Hamilton, Mark

Hanseeuw, Bernard

Hansen, Niels

Hanson, J.

Harada, Kouji

Harbison, Joe

Harbo, Thomas*

Hardy, Todd A.

Haroche, Julien

Hasan, D.

Hashemolhosseini, Said

Hauck, Erik

Hautecoeur, Patrick

He, Qijun

Hedberg, P.

Hellwig, Kerstin

Hemmer, Bernhard

Hernandez Fustes, Otto Jesus

Hertz, Daniel L.

Heusch, Gerd

Hewamadduma, Channa

Hillert, Jan

Hirosawa, Takanobu

Hodge, Allison M.

Hoeftberger, Romana

Hoepner, Robert

Hoffmann, Sarah

Hofso, Kirstin

Hogeveen, Lara

Holleman, J.

Holmøy, Trygve

Höltje, Markus

Holtz, Anna‐Victoria

Hong, Zhen

Hori, Satoshi

Horowitz, Tatiana

Hou, Shuai

Houlden, Henry

Hu, Wei Syun

Huda, Saif

Hussain, Yessar

Icoz, Mehmed

Ihara, Masafumi*

Inamasu, Joji

Inan, Levent

Intiso, Domenico

Iodice, Rosa

Iorio, Raffaele*

Ishikawa, Masatsune*

Ishizuka, Kentaro

Iskrov, Georgi

Isohanni, Pirjo

Iwasa, Kazuo

Jabbarli, Ramazan*

Jacobi, Heike

Jagannathan, Ram

Jakob Brecl, Gregor

Janes, Francesco

Jatužis, Dalius

Jauss, Marek

Jayalakshmi, Sita

Jeenduang, Nutjaree

Jensen, Rigmor

Ji, Xunming

Jia, Xiangjie

Johansson, Martin

Jokinen, Hanna

Jolkkonen, Jukka

Jonas, Yeung

Joubert, Bastien

Juega, Jesús

Jumaa, Mouhammad

Kaasinen, Valtteri

Kafaie, Jafar

Kakaletsis, Nikolaos

Kalaria, Raj

Kalincik, Tomas

Kallweit, Ulf

Kaminski, Henry

Kamm, Christoph

Kan, Hermien E.

Kana, Veronika

Kantanen, Anne‐Mari

Kaplan, Tamara

Karapanayiotides, Theodoros

Karlsson, Pall

Karnon, Jon

Karsan, Nazia

Kaski, Diego

Katsuno, Masahisa

Kaushik, Kanishk

Kearney, Hugh

Kelly, Mark

Khairnar, Amit

Khoo, Ching Soong

Khosravani, Houman

Kiernan, Matthew

Kiersnowski, Oliver

Killestein, Joep

Kim, Jong

Kim, Joong‐Seok

Kimura, Akio

Kister, Ilya

Klapproth, Susan

Klein, Ainat

Klein, Piers

Kleopa, Kleopas

Klimkowicz‐Mrowiec, Aleksandra

Klingebiel, Randolf

Klockgether, Thomas

Klomjai, W.

Knake, Susanne

Kneihsl, Markus

Knier, Benjamin

Knoerl, Robert

Koemans, Emma

Koga, M.

Kohle, Felix

Koike, Haruki**

Kokotis, Panagiotis

Korinthenberg, Rudolf

Kosutzka, Zuzana

Kowacs, Pedro

Krarup, Christian

Kremer, Christine

Kringler, Wolfgang

Krishnamoorthy, Soumya

Krogias, Christos

Krommyda, Magdalini

Kruja, Jera

Kruuse, Christina

Kümpfel, Tania

Kunicki, Zachary

Kuppuswamy, A.

Kurmi, Om P.

Kurth, Ingo

Kusunoki, Susumu

Kuwabara, Satoshi

Kwan, Park

Kytövuori, Laura

Labeit, Bendix

Labeyrie, Céline

Laine, Matti

Lainez, Miguel

Lakhani, Da.

Lambert, Nicolas

Landi, Doriana

Lang, Anthony

Lange, Kristin Sophie

Larner, Andrew

Laton, Jorne

Laugesen, Nicolaj

Leal Rato, Miguel

Lebedeva, Elena**

Lebrun‐Frenay, Christine

Ledwon, Daniel

Lee, Jee‐Young

Lee, Kyung Bok

Lee, Richard E.

Lee, Wan‐Ping

Lee, Yi‐Chu

Lehmann, Helmar

Lehn, Alexander

Lehtisalo, Jenni

Leira, Yago

Leite, Maria Isabel

Lemmens, Robin

Leocani, Letizia

Leone, C.

Leray, Emmanuelle

Lesca, Gaetan

Lesourd, Mathieu

Leta, Valentina

Lewis, Steven

Ley, Sylvia

Li, Hao

Li, Tong

Li, Xiao‐Guang

Li, Xu

Liao, Yi‐Chu

Libonati, Laura

Libório, Alexandre Braga

Lidstone, Sarah

Liewluck, Teerin

Lin, Hsien‐Ho

Linn, Jennifer

Lioutas, Vasileios‐Arsenios**

Litwin, Tomasz

Liu, Chen

Liu, Siwei

Liu, Xiaodan

Llorens, Roberto

Lo, Eng

Lochner, Piergiorgio

Loewen, Peter Loewen

Logroscino, Giancarlo*

Long, Youming

Longo, Miriam

Longo, Nicola

López‐Sendón, Jose

Lorefice, Lorena

Lorscheider, Johannes

Lotan, Itay

Louis, Elan

Lozeron, Pierre

Luis Guerrero‐Peral, Ángel*

Lunetta, C.

Ma, Guolin

Maas, Andrew

Macaron, Gabrielle

Macerollo, Antonella

Macerollo, Antonella

Macey, Paul

Macías‐García, Daniel

Macleod, Angus

Maclver, Claire

Maeda, Takuma

Maeda, Tetsuya

Maestri, Michelangelo

Maestrini, Ilaria

Magalhães, Andreia

Maggi, Lorenzo

Magyari, Melinda

Malfatti, Edoardo

Malik, Rayaz

Malojcic, Branko

Mancuso, Michelangelo*

Manera, Umberto

Mangiardi, Marilena

Manuello, J.

Marano, Massimo

Margoni, Monica

Mariotto, Sara**

Marquetand, Justus

Marriott, James

Martino, Davide

Marto, João

Mathiesen, Tiit

Mathon, Bertrand

Matias‐Guiu, Jordi A.

Matsuo, Ryu

Mattioli, Irene

May, Arne

Mayer, Geert

Mazidi, Mohsen

Mazighi, Mikael

Mccombe, Pamela A.

Mcgovern, Eavan

Mcgrath, Stephanie

Mchutchison, Caroline

Mckeon, Andrew

Mcmackin, R.

Mecocci, Patrizia

Meisel, Andreas

Meles, Sanne K.

Meletti, Stefano

Mencacci, Niccolo

Menelaos, P.

Menzler, Katja

Merlino, Giovanni

Messina, Roberta

Meyer, Thomas

Miatton, Marijke

Michel, Laure

Michels, Dm.

Mills, Kelly

Minami, Kisei

Mioshi, Eneida

Misra, Usha**

Mitrea, Dan

Moccia, Mrcello

Moeller, Harald

Moisset, Xavier

Moloney, Patrick

Morel, Eric

Morgante, Francesca

Moro, Elena

Moroni, Isabella

Morotti, Andrea

Moscote‐Salazar, Luis

Moskau‐Hartman, Susanna

Moulin, Solène

Moulin, Thierry

Muhammad, Bilal

Müller, Jannis

Muñoz‐Delgado, Laura

Murphy, Sinead

Navarro Pérez, María Pilar

Nemoto, Kiyotaka

Neuteboom, Rinze

Nevo, Yoram

Nezu, Tomohisa

Ng, Felix C.

Nicolas, Gaël

Nicolini, Ettore

Nielsen, Rune

Nikaido, Yasutaka

Nishiyama, Masahiro

Nobile‐Orazio, Eduardo

Nurmikko, Turo

Nyholm, Dag

Nyquist, Paul

O'connor, Kevin

Oerlemans, Joyce

Ogasawara, K.

O'grady, Kristin

Ogrinc, Katarina

Okazaki, Shuhei

Okuda, Darin

Olivot, Jean Marc

Ong, Pei Shi

Ornello, Raffaele

Ortiz‐Prado, Esteban

Oterdoom, D. L. Marinus

Oudit, Gavin

Ozdinler, Hande

Ozturk, Serefnur

Pagano, Paolo

Palaiodimou, Lina

Palandri, Giorgio

Palavra, Filipe

Palazzo, Paola

Pandey, Sanjay

Pantoni, Leonardo*

Parchi, Piero

Pareyson, Davide*

Park, Chae Jung

Park, Kang Min

Parry‐Jones, Ar

Pasi, Marco

Pasqualotto, Eric

Pathak, Abhishek

Pathan, Faraz

Pavsic, Katja

Payabvash, Seyedmehdi

Payne, Stephen

Peattie, Alexander

Pedrosa, David

Pellecchia, M. T.

Pellesi, Lanfranco

Pelosi, Luciana

Pengo, Marta

Perera, A.

Perez, David

Perić, Stojan

Perin, Cecilia

Perry, David

Petkus, Andrew

Petri, Susanne

Petzold, Axel

Pezzini, Alessandro

Pfeuffer, Steffen

Phenwan, Tharin

Picca, Alberto

Pichon, Maud

Picietti, Edoardo

Picillo, Marina

Pico, Fernando

Piehl, Fredrik**

Pilato, Fabio

Pilotto, Andrea

Pinčáková, Katarína

Pinho, João**

Pinter, Daniela

Pinto, Susana

Piper, Rory

Pires, Luís

Pitarokoili, Kalliopi*

Pitteri, Marco

Pizzorni, Nicole

Plant, Gordon

Podnar, Simon

Popkirov, Stoyan

Portaccio, Emilio

Power, David A.

Prabhakar At.

Prapiadou, Savvina

Prats‐Sánchez, Luis

Preethish‐Kumar, Veeramani

Premi, Enrico

Prosperini, Luca*

Provenzano, Carlo

Prust, Morgan L.

Psychogios, Marios

Punga, Anna

Purroy, F.

Putaala, Jukka

Qiu, Wei

Quek, Amy May Lin

Querin, Giorgia

Querol, Luis

Quinn, Terry

Radakovic, Ratko*

Radojewski, Piotr

Raffaelli, Bianca

Raggi, Alberto

Ragonese, Paolo

Rahouma, Mohamed

Raj, Satish R.

Rajabally, Yusuf A.**.

Ramage, Emily

Ramanathan, Murali

Ramanujam, Bhargavi

Ramdas, Sithara

Ranta, Anna

Rass, Verena

Rätsep, Tõnu

Rattay, Tim

Rautalin, Ilari

Ravits, John

Regensburger, Martin

Rêgo, André

Rehmann, Robert

Reichmann, Heinz

Reilly, Mary

Reimão, Sofia

Rejdak, Konrad

Rektorova, Irena

Remiche, Gauthier*

Reyes, Alvaro

Rezaei, Zahra

Rhomberg, Thomas

Riazi, Sheila

Ricci, Stefano

Ricciardi, Lucia

Rice, Claire

Richard, Sébastien

Richardson, Jason R.

Richfield, Edward

Riek, Heidi

Riley, Claire

Ripellino, Paolo

Ritzel, Rodney

Riva, Nilo

Rivier, Cyprien

Rizos, Timolaos

Rizzo, Giovanni

Roberts, Nicole

Robinson, Gail

Rocca, Maria

Rodrigues Silva, Pedro

Rodriguez‐Labrada, Roberto

Romagnolo, Alberto

Romano, Jose

Romigi, Andrea

Rosa, Mário

Rossi, Simone

Rostasy, Kevin

Rostedt Punga, Anna

Roux, Alexandre

Royl, Georg

Ruban, Angela

Rückert, Jens

Rudnik‐Schoeneborn, Sabine

Ruigrok, Y. M.

Rukavina, Katarina

Ruprecht, Klemens

Rutten, Julie

Ruuskanen, Jori

Ryan, Joanne

Rzepiński, Łukasz

Sabanathan, Sara

Saccà, Francesco

Sacco, Simone

Sacre, Karim

Sairanen, Tiina

Sakakibara, Ryuji

Salhofer‐Polanyi, Sabine

Salter, Amber

Salvadori, Emilia

Salvalaggio, Alessandro

Samaniego, E. A.

San‐Juan, Daniel

Santamaria, Joan

Santorelli, Filippo M.

Santos Santos, Miguel

Santos‐Lasaosa, Sonia

Sara, Cabras

Sarkis, Rani A.

Sasaki, Nozomi

Sasegbon, Ayodele

Satolli, Sara

Saute, Jonas

Scalfari, Antonio

Schankin, Christoph

Schenck, C. H.

Schiehser, Dawn

Schirinzi, Tommaso

Schmid, Annina B. B.

Schnakers, Caroline

Schöberl, Florian

Schofield, Jill R.

Schoonheim, Menno

Schoser, Benedikt**

Schwab, Nicholas

Schwarzenhofer, Daniel C.

Schwingenschuh, P.

Scopelliti, Giuseppe

Scutelnic, Adrian

Sechi, Elia

Sedley, William

Seeman, Pavel

Seiffge, David

Sejbaek, Tobias

Sekula, Raymond F.

Sellner, Johann***

Sen, Souvik

Seners, Pierre

Sensi, Mariachiara

Serebruany, Victor

Serra, Carlo

Servadei, Franco

Sewry, Caroline

Shah, Vrutangkumar

Shahrizaila, Nortina

Shalash, Ali

Shimoyama, Takashi

Shindo, Akihiro

Shrestha, Srishti

Shy, Michael

Sibon, Igor

Siepmann, Timo*

Signori, Alessio*

Simpson‐Yap, Steve

Siuda, Joanna

Smid, Jerusa

Smilowska, Katarzyna

Smith, Mary Lou

Smyth, Duncan

Sokolov, Arseny

Solheim, Anne

Solje, Eino

Soman, Salil

Sopranzi, Federico

Soto‐Rodríguez, Francisco Javier

Southerland, Andrew

Souza, Paulo Victor

Spillane, Jennifer

Spinelli, Edoardo

Stefani, Ambra

Stefanis, Leonidas

Steiner, Leon Amadeus

Stenzel, Werner

Sterenstein, Andrea E.

Stojkovic, Tanya

Storti, Benedetta

Straube, Andreas

Strijbis, Eva

Strle, Franc

Strunk, Daniel

Stuifbergen, Alexa

Stupica, Daša

Sun, Dianjianyi

Sun, Jianjun

Sunnerhagen, Katharina Stibrant

Suzuki, Keisuke***

Sveikata, Lukas

Sveinsson, Olafur Arni

Svenningsson, Anders

Svenningsson, Per

Swinnen, Bart

Taba, Pille

Taipa, Ricardo

Takeda, Takahiro

Takizawa, Tsubasa

Talelli, Pinelopi

Tambasco, Nicola

Tan, Cheng‐Yin

Tanasescu, Radu**

Tang, Whitney

Tankisi, Hatice**

Tannemaat, Martijn

Tao, Yingqun

Tarnutzer, Alexander

Taso, Manuel

Tassorelli, Cristina

Tavares‐Júnior, José Wagner

Telleman, Johan

Teodoro, Tiago

Ter Telgte, Annemeike

Terhart, Maria

Terwindt, Gisela

Tessitore, Alessandro

Teunissen, Charlotte

Theodorou, Aikaterini

Theys, Tom

Thiankhaw, Kitti

Thieme, Andreas

Thijs, Roland

Thijs, Vincent

Thirugnanam, Umapathi N.

Tilsley, Peneolope

Timpani, Cara

Timsit, Serge

Titcomb, Tyler

Todea, Ramona‐Alexandra

Togo, Masaya

Tomaselli, Pedro

Tomic, Svletana

Topiwala, Karan K.

Torrente, Angelo

Tosatti, Jessica

Toscano, Antonio

Traenka, Christopher

Tremblay, Josh

Trimouille, Aurelien

Trojsi, Francesca

Truini, Andrea

Tsai, Hsin‐Hsi

Tsivgoulis, Georgios**

Tsuji, Shoji

Tullberg, Mats

Turner, Martin

Tuttolomondo, Antonino

Tyvaert, Louise

Uchida, Yuto

Uchino, Ken

Ueno, Yuji

Uher, Tomas**

Uncini, Antonino**

Urra, Xabier

Urso, Daniele

Utianski, Rene

Uwishema, Olivier

Valente, Enza Maria

Valente, Kette

Valenzuela, Antonia

Van Damme, Philip

Van De Warrenburg, Bart P.

Van Den Berg, S. A.

Van Der Kooi, Anneke

Van Der Meer, Lucienne B.

Van Der Mei, Ingrid

Van Der Worp, H. B.

Van Doorn, Pieter A.

Van Eijk, Jeroen

Van Rooij, Willem Jan

Van Veelen, Nancy

Vanbellingen, Tim

Vandebergh, Marijne

Vandenberghe, R.

Vandenberghe, Rik

Vanegas, Maria

Vanopdenbosch, Ludo

Vanwaarde, Jeroen

Vanweehaeghe, Donatienne

Vassilaki, Maria

Veeramuthu, Vigneswaran

Verde, Federico

Verghese, Joe

Vergouwen, Mervyn

Vermersch, Patrick

Vernieri, Fabrizio

Viader, Fausto

Vial, Felipe

Viana, Michele

Vidal, Julie

Viggiano, Davide

Villanueva, Vicente

Vincent, Angela

Vineet, Punia

Vissing, John

Von Wyl, Viktor

Voordouw, Maarten

Vucic, Steve

Vukusic, Sandra

Vuletic, Vladimira

Vyas, Manav V.

Wahlster, Sarah

Waldemar, Gunhild

Waliszewska‐Prosol, Marta**.

Walter, Maggie

Walter, Silke

Wandscher, Kathrin

Wang, Anxin

Wang, Tao

Weidner, Norbert

Weis, Joachim

Weiss, Nicolas

Weissenborn, Karin

Werner, Ralph

Werring, David

Westeneng, Henk‐Jan

Weydt, Patrick

Wing, Yun‐Kwok

Winther Schytz, Henrik

Wischmann, Johannes

Wöber, Christian

Wolf, Katharina

Wolf, Nicole

Wong, C.

Wong, Joseph

Wong, Pei Shieen

Woodward, Michael

Wu, Songdi

Xu, Hong

Yamamoto, Tatsuya**

Yang, Lin

Yang, Xu

Yassuda, Monica Sanches

Ye, Byoung Seok

Yeni, Kubra

Yin, Zhijun

Yoshida, H.

You, Jason

Yu, Amy

Zakrzewska, J. M.

Zandi, Michael

Zawadka‐Kunikowska, Monika

Zedde, Marialuisa**

Zegers, Karen

Zelano, Johan

Zhang, Aihua

Zhao, Bing

Zhao, Henry

Zhao, Jingcheng

Zheng, Peng

Zheng, Qin

Zhu, Chengcheng

Ziai, Wendy C.

Zimprich, Fritz

Zoccolella, Stefano

Zou, Zhangyu

